# Brain gray matter structural network in myotonic dystrophy type 1

**DOI:** 10.1371/journal.pone.0187343

**Published:** 2017-11-02

**Authors:** Atsuhiko Sugiyama, Daichi Sone, Noriko Sato, Yukio Kimura, Miho Ota, Norihide Maikusa, Tomoko Maekawa, Mikako Enokizono, Madoka Mori-Yoshimura, Yasushi Ohya, Satoshi Kuwabara, Hiroshi Matsuda

**Affiliations:** 1 Department of Radiology, National Center of Neurology and Psychiatry, Tokyo, Japan; 2 Department of Neurology, Graduate School of Medicine, Chiba University, Chiba, Japan; 3 Integrative Brain Imaging Center, National Center of Neurology and Psychiatry, Tokyo, Japan; 4 Department of Mental Disorder Research, National Institute of Neuroscience, National Center of Neurology and Psychiatry, Tokyo, Japan; 5 Department of Neurology, National Center of Neurology and Psychiatry, Tokyo, Japan; University of Modena and Reggio Emilia, ITALY

## Abstract

This study aimed to investigate abnormalities in structural covariance network constructed from gray matter volume in myotonic dystrophy type 1 (DM1) patients by using graph theoretical analysis for further clarification of the underlying mechanisms of central nervous system involvement. Twenty-eight DM1 patients (4 childhood onset, 10 juvenile onset, 14 adult onset), excluding three cases from 31 consecutive patients who underwent magnetic resonance imaging in a certain period, and 28 age- and sex- matched healthy control subjects were included in this study. The normalized gray matter images of both groups were subjected to voxel based morphometry (VBM) and Graph Analysis Toolbox for graph theoretical analysis. VBM revealed extensive gray matter atrophy in DM1 patients, including cortical and subcortical structures. On graph theoretical analysis, there were no significant differences between DM1 and control groups in terms of the global measures of connectivity. Betweenness centrality was increased in several regions including the left fusiform gyrus, whereas it was decreased in the right striatum. The absence of significant differences between the groups in global network measurements on graph theoretical analysis is consistent with the fact that the general cognitive function is preserved in DM1 patients. In DM1 patients, increased connectivity in the left fusiform gyrus and decreased connectivity in the right striatum might be associated with impairment in face perception and theory of mind, and schizotypal-paranoid personality traits, respectively.

## Introduction

Myotonic dystrophy type 1 (DM1) is the most common form of inherited muscular dystrophy in adults [1]. It is caused by the expansion of CTG repeats in the 3’-untranslated region of the *myotonic dystrophy protein kinase* (*DMPK*) gene on chromosome 19 [2]. In addition to characteristic neuromuscular symptoms such as limb muscle weakness and myotonia, DM1 affects various organs and systems, including some in the central nervous system (CNS) [3]. Cognitive deficits are common manifestations in patients with DM1 and are characterized by defective facial memory and facial emotion recognition, executive, attentional, memory, and visuospatial dysfunction [4–9]. Daytime sleepiness, fatigue, impairment in odor recognition and theory of mind, and specific personality traits have been also reported in DM1 [5, 10, 11].

Several reports have documented brain abnormalities possibly associated with these CNS symptoms, such as white matter lesions on conventional MR imaging [12–14], regional gray matter volume reduction on voxel based morphometry (VBM) [7, 14–21], white matter damage on diffusion tensor imaging [14, 16, 17, 19–21], and reduced blood flow and hypometabolism on molecular imaging (single-photon emission computed tomography and positron emission tomography) [7, 12, 22]. Although the mechanism of CNS involvement in DM1 has not been fully elucidated, some of these studies suggested a disconnection of cortical regions, secondary to white matter tract breakdown, as a potential mechanism [14, 17, 21].

Several methods are used to investigate brain connectivity in neurological and psychiatric diseases. Recently, graph theoretical analysis has been introduced as a powerful method for quantifying and characterizing brain networks [23]. Graph theoretical analysis is a mathematical method that enables the analysis of connectivity networks by applying it to brain images or electrophysiology. Numerous studies have illustrated alteration of arrangements in structural and functional brain networks in normal development, aging, and neurological and psychiatric disorders by applying graph theoretical analysis to MR imaging [24]. Brain structural covariance analysis has been proposed as a valid measure to infer large-scale structural brain networks, and there have been multiple graph theoretical studies seeking to assess structural correlation networks constructed from regional gray matter volume in brain disorders [25, 26]. In DM1 patients, a previous study of the graph theoretical analysis of resting-state functional MRI has shown reduced connectivity in a large frontoparietal network and its correlation with impairment in visuospatial reasoning [27]. However, to date, there has been no report using graph theoretical technique for structural MR imaging in DM1 patients. This study was conducted to investigate abnormalities in structural covariance networks constructed from gray matter volume in DM1 patients using graph theoretical analysis with the aim to further clarify the mechanism of CNS involvement.

## Materials and methods

### Subjects

In total, 31 consecutive genetically confirmed DM1 patients who underwent brain MRI from June 2014 to December 2016 at our institution were identified in our database. Brain MRI was performed in DM1 subjects to evaluate CNS involvement at the time of diagnosis, admission for evaluation of complications, and during follow-up in the out-patient clinic. Two patients were excluded because of lesions due to another etiology (one with a mass in the midbrain, the other with brain contusion). An additional patient was excluded because of missing information on CTG repeat length. Finally, 28 DM1 patients and 28 age- and sex-matched healthy controls who volunteered in response to advertisements were included in this study. DNA was extracted from peripheral lymphocytes and analyzed for expansion of the CTG repeat in the *DMPK* gene. Medical records of the 28 DM1 patients were reviewed for disease duration, age at onset, age at scan, age at analysis for the CTG repeat, mini-mental state examination (MMSE), and muscular impairment rating scale (MIRS). MMSE was examined in 21 of the 28 DM1 patients, and a score of 24 was taken as the cut off for MMSE. This retrospective study was approved by the institutional review board at the National Center of Neurology and Psychiatry Hospital, and the need for patient informed consent was waived.

### MRI data acquisition and processing

All subjects were scanned using a 3.0-tesla MR system with a 32-channel coil, and 20 DM1 patients and 20 healthy controls were scanned using a Verio 3.0-tesla MRI scanner (Siemens Medical System, Erlangen, Germany). The parameters of three-dimensional T1-weighted magnetization prepared rapid acquisition with gradient echo (MPRAGE) images were as follows: repetition time/echo time: 1800 ms/2.26 ms, 288 × 320 matrix, field of view 25 × 25 cm, number of excitations: 1, 0.8 mm with no interslice gap, and 224 continuous sagittal slices. Eight DM1 patients and eight healthy controls were scanned using an Achieva 3.0-tesal MR imaging scanner (Philips Medical Systems, Best, The Netherlands). The parameters of three-dimensional T1-weighted MPRAGE images were as follows: repetition time/echo time: 7.12 ms/3.4 ms, matrix of 260×320, field of view 26×24 cm, number of excitations: 1, 0.6 mm with no interslice gap, 300 sagittal slices.

### VBM analysis

VBM analyses were performed using Statistical Parametric Mapping software (SPM12: Wellcome Trust Centre of Neuroimaging, London, UK) running in Matlab 2014a (The Mathworks, Natick, MA, USA). First, three-dimensional T1-weighted MPRAGE images were segmented in gray matter (GM), white matter (WM), and cerebrospinal fluid using the standard unified segmentation model in SPM12. These segmented GM and WM images were spatially normalized to a customized template in standardized anatomic space using the diffeomorphic anatomical registration through exponentiated lie algebra (DARTEL) method. Each image was then modulated and spatially smoothed with an 8-mm full-width at half-maximum Gaussian Kernel.

We compared regional GM volume between groups using a two-sample *t* test, with age, sex, and type of MRI system as covariates. The correlation between regional GM volume and number of CTG repeats, disease duration, age at onset, and MIRS in DM1 patients was assessed using SPM12 including age, gender, and type of MRI system as covariates. The results were explored at a false discovery rate-corrected threshold of *p* <0.05 at peak-level.

### Graph theoretical analysis

Graph theoretical analyses were performed on the regional covariance matrices using the Graph Analysis Toolbox (GAT) [28]. BrainNet Viewer was used for visualization of regional analyses [29]. The normalized GM images of DM1 patients and healthy controls were applied to GAT running in MATLAB 2014a with age and gender as nuisance covariates. Global scaling was used to correct for global atrophy. The 90 cortical and subcortical regions of interest (ROIs) from the Automated Anatomical Labeling template were used as the regional parcellation scheme [30].

For construction of the structural correlation network, GAT analyses of all 90 ROIs and 90 × 90 association matrices for DM1 and control groups were generated using the Pearson correlation coefficient. Binary adjacency matrices were then derived by thresholding association matrices at densities ranging from 0.10 to 0.50 at intervals of 0.02. Network hub analysis was also performed, and a node was considered as a hub if its betweenness centrality (BC) was 1 SD higher than the mean betweenness. The BC is a measure of the number of shortest paths that traverse a given node and is used to detect nodes that are highly central for important anatomical or functional connections.

Then, the two key metrics, clustering coefficient (*C*), and characteristic path length (*L*), were calculated. The clustering coefficient is a measure of the number of edges that exist between its nearest neighbors. The characteristic path length is the average shortest path length between all pairs of nodes in the network, and it is used as a measure of network integration. The small-world-ness was calculated as [C/C_rand_]/[L/L_rand_] where C_rand_ and L_rand_ are the mean clustering coefficient and the characteristic path length of 20 random networks, respectively. The following network metrics were also calculated: global efficiency; the exchange of information across the whole network, which is inversely related to the path length; local efficiency; the inverse of the average shortest path connecting neighbors of nodes; assortativity, a measure of preference for network’s nodes to attach to others that are similar; transitivity, a measure of network segregation; and modularity, a measure of the strength of division of a network into modules.

Moreover, network resilience to random failure and to targeted attack was evaluated. Network resilience to random failure was assessed by randomly removing one node from the network and then measuring changes in global metrics of the remaining largest component, and this process was repeated by removing additional nodes randomly until the size of the largest component reached one. The network resilience to targeted attack was assessed by removing nodes in rank order of decreasing nodal BC.

For regional network analyses, GAT was used to perform nonparametric permutation tests and assess regional differences in BC and clustering of connectivity between the DM1 and control groups for each comparison.

### Statistical analyses

All statistical analyses were performed using the SPSS software, ver. 22.0 (SPSS Japan, Tokyo). Demographic variables were analyzed using the Student’s t-test for continuous variables and the chi-squared test for categorical variables. A *p* value less than 0.05 was considered statistically significant.

GAT compared the areas under a curve (AUC) of each network measure and resilience of the DM1 group to those of the control group. To test the significance, the actual between-group difference in AUC for each network measure was placed in the corresponding permutation distribution, and two-tailed *p*-values were calculated on the basis of their percentile position. In addition, GAT conducted a one-tailed nonparametric permutation test with 1000 repetitions to evaluate the regional differences in BC and clustering between DM1 and control groups. To correct for multiple comparisons, the false discovery rate of *p* <0.05 was considered significant.

## Result

### Demographics

Demographic information and clinical data of the participants are shown in Table 1. No significant differences in age and sex were observed between control and DM1 patients. The median MMSE score was 28 (range, 13–30) for the 21 DM1 patients and 30 (range, 26–30) for the 28 healthy controls. Four (three juvenile onset, one adult onset) of the 21 DM1 patients showed decreased MMSE scores. Details of the clinical characteristics of the DM1 patients are listed in S1 Table.

**Table 1:** Demographic and clinical data of patients with myotonic dystrophy type 1 and healthy subjects.

	DM1 patients (n = 28)	Healthy subjects (n = 28)	*p* value
Sex (Male/Female)	16/12	16/12	NS
Age at MRI, y (mean±SD)	42.5 ± 11.2	43.4 ± 10.0	NS
Age at onset, y (mean±SD)	20.5 ± 8.5	NA	NA
Age at onset, No. (%) of patients			
Congenital	0	NA	NA
Childhood (age range, 1-10y)	4 (14)	NA	NA
Juvenile (age range, 11-20y)	10 (36)	NA	NA
Adult (age range, 21-40y)	14 (50)	NA	NA
Late adult (age range, 41-60y)	0	NA	NA
Age at analysis for the CTG repeat, y (mean±SD)	37.1 ± 10.7	NA	NA
Disease duration, y (mean±SD)	22.0 ± 12.4	NA	NA
CTG repeat length (median [range])	662.5 (133–3000)	NA	NA
Expansion group, No. (%) of patients			
E1 (50–150)	1 (4)	NA	NA
E2 (151–500)	7 (25)	NA	NA
E3 (501–1000)	14 (50)	NA	NA
E4 (>1000)	6 (21)	NA	NA
MIRS score, No. (%) of patients			
1	0	NA	NA
2	1 (4)	NA	NA
3	2 (7)	NA	NA
4	22 (79)	NA	NA
5	3 (11)	NA	NA

DM1, myotonic dystrophy type 1; NS, not significant; MRI, magnetic resonance imaging; SD, standard deviation; NA, not applicable; MIRS, muscular impairment rating scale

### Volumetric analysis

**Fig 1:**
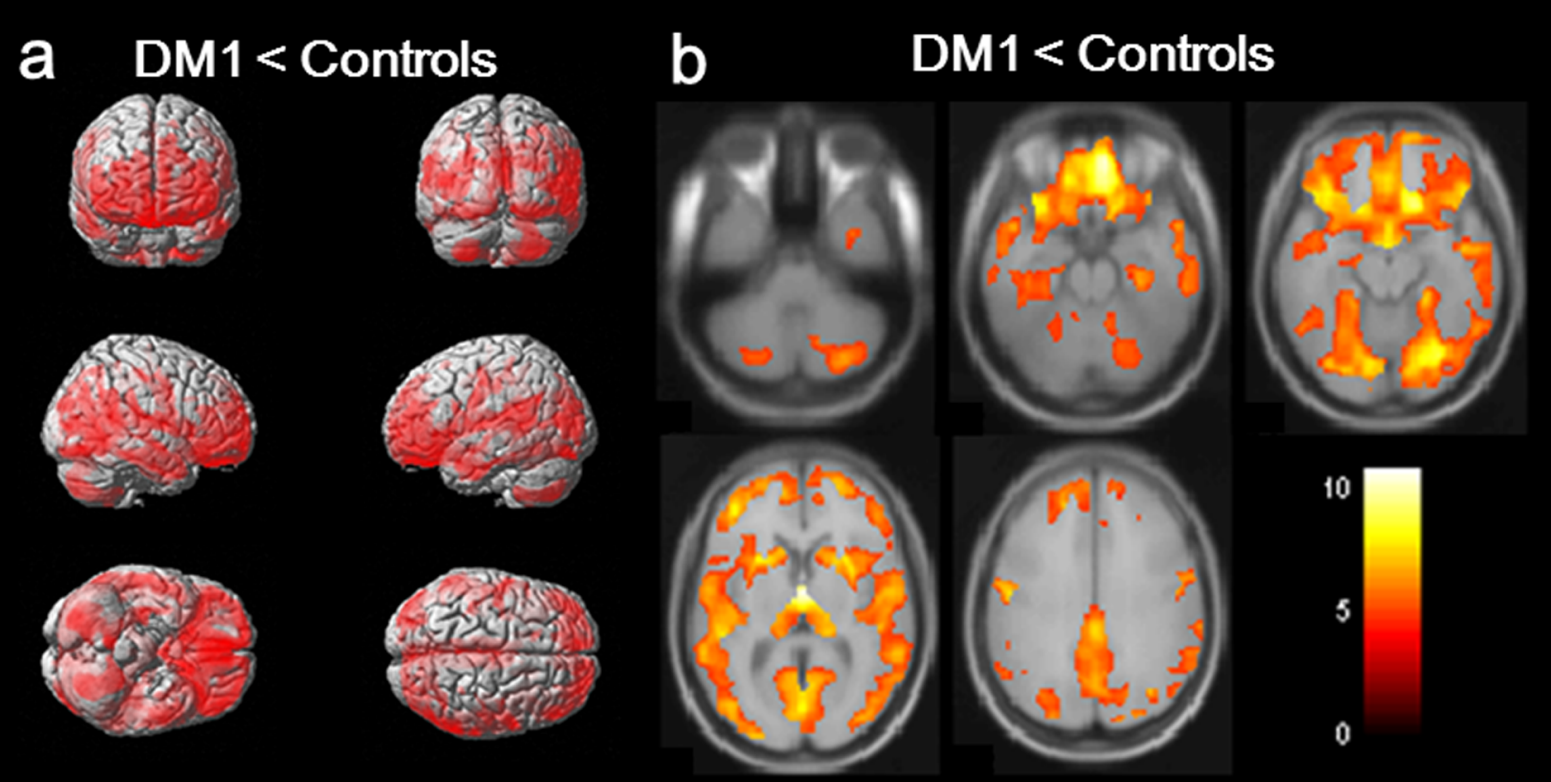
Regional gray matter (GM) atrophy in myotonic dystrophy type 1 (DM1) patients compared with controls. Clusters of reduced GM volume in DM1 patients compared with controls (clusters significant at *p* <0.05, FWE corrected, extended threshold = 49 voxels). The colored bar represents the T score. Clusters are diffuse in both hemispheres, particularly in the orbitofrontal, medial frontal, precentral, anterior insula, posterior cingulate, superior temporal, hippocampal, parahippocampal, fusiform, and lingual areas. Clusters are also found bilaterally in subcortical areas, including the thalamus, caudate nucleus, and putamen.

VBM revealed almost symmetric GM atrophy in DM1 patients compared to controls, including cortical and subcortical structures (Fig 1). Particularly, orbitofrontal, medial frontal, precentral, anterior insular, posterior cingulate, superior temporal, hippocampal, parahippocampal, fusiform, and lingual areas were affected bilaterally. Subcortical GM atrophy was also found in the thalamus, caudate nucleus, and putamen bilaterally. There was no significant correlation between regional GM volume and CTG repeat length, disease duration, age at onset, and MIRS.

### Graph theory analysis

**Fig 2:**
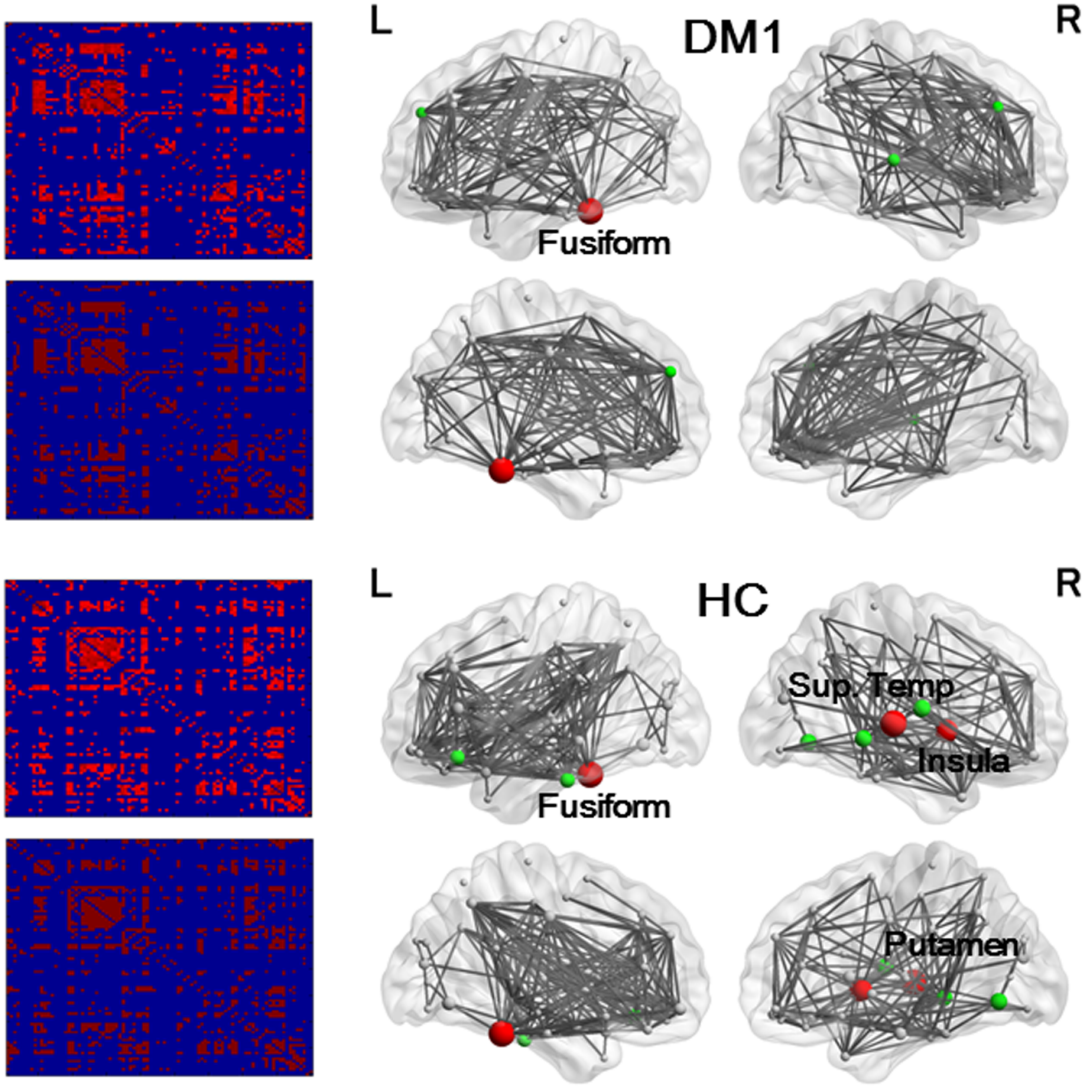
Results of association matrices (left) and network hub nodes and edges (right). Lines indicate the edge, and spheres represent nodes. The size of the nodes is proportional to the betweenness centrality (BC). Red denotes hub nodes with BC > 2 standard deviation (SD), and green denotes BC > 1 SD. In DM 1 patients, hub nodes with BC > 2 SD were found only in the left fusiform gyrus, and hub nodes with BC > 1 SD were found in the superior and middle frontal gyrus, and superior temporal gyrus. In controls, hub nodes with BC > 2 SD were found in the right insula, putamen, superior temporal gyrus, and left fusiform gyrus, and hub nodes with BC > 1 SD were found in the middle and inferior gyrus, orbitofrontal gyrus, lingual gyrus, and rolandic operculum.

Inter-regional cortical brain regions correlation matrices of controls and DM1 patients are shown in Fig 2.

**Fig 3:**
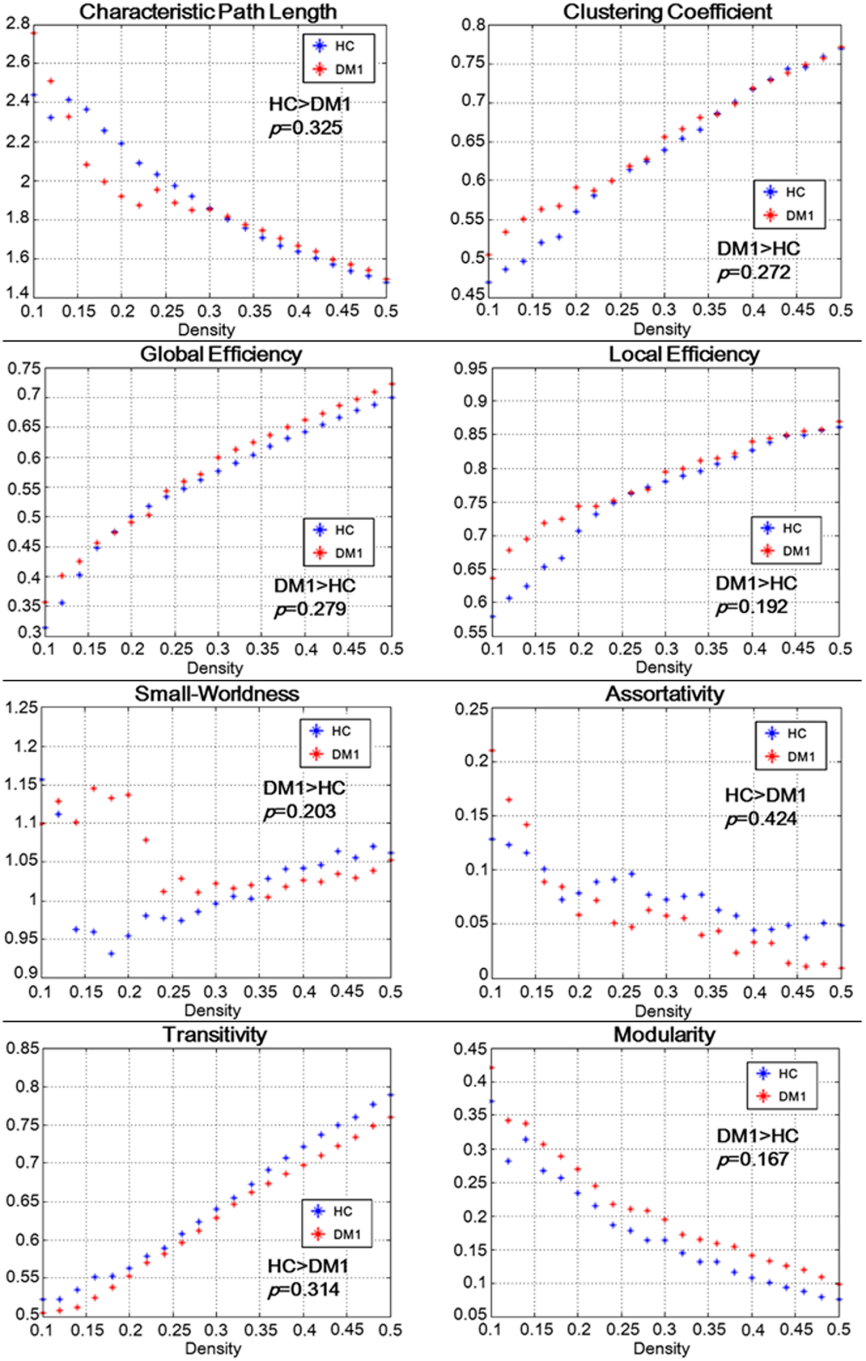
Results of network metrics and the p-values of AUC comparisons between DM1 and controls. No significant differences were found for measures of global and local network organization (characteristic path length, clustering, efficiency, small-worldness, assortativity, transitivity, and modularity) between DM1 patients and controls.

In the network metrics, no significant differences were found for measures of global and local network organization (small-world-ness, characteristic path length, efficiency, modularity, transitivity, assortativity, clustering) between controls and DM1 patients (Fig 3). There were no significant differences in the network resilience to either random or targeted attack between controls and DM1 patients (S1 Fig).

Hub regions and edges in the two groups are shown in Fig 2. While hub nodes with BC values > 2 SD were found in the right insula, middle temporal gyrus, putamen, and left fusiform gyrus in controls, these were only found in the left fusiform gyrus in DM1 patients.

**Fig 4:**
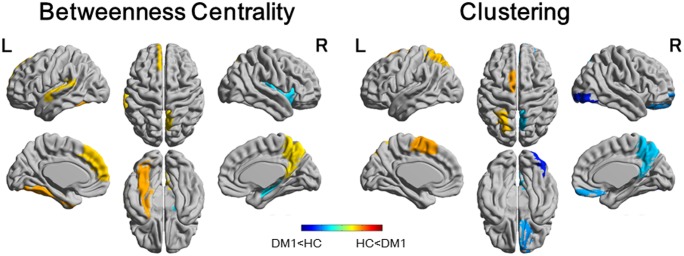
Regional comparison of myotonic dystrophy type 1 (DM1) group with healthy control (HC) group for betweenness centrality (BC) and clustering. Colored areas depict significant differences (at a false discovery rate of *p* < 0.05). In DM1 patients, an increased BC was found in the left fusiform gyrus, superior temporal gyrus, superior frontal gyrus, and right precuneus, and a decreased BC was found in the right caudate nucleus and putamen. Comparison of clustering showed decreased regions in the right orbitofrontal gyrus, inferior occipital gyrus, precuneus, left pallidum, and increase regions in the left supplementary motor area and superior parietal lobe.

Fig 4 illustrates the regional differences of BC and clustering in DM1 patients compared to the controls. The DM1 patients showed increased BC in the left fusiform gyrus, superior temporal gyrus, superior frontal gyrus, and right precuneus. The DM1 patients also showed decreased BC in the right caudate nucleus and putamen. The comparison of clustering showed decreased regions in the right orbitofrontal gyrus, inferior occipital gyrus, precuneus, left pallidum, and increased regions in the left supplementary motor area and superior parietal lobe.

## Discussion

The present study is the first to explore GM volume covariance network in DM1 patients using graph theoretical analysis. In contrast to the wide range of GM volume reductions in DM1 patients on VBM, there were no significant differences between DM1 and control groups in global measures of connectivity on graph theoretical analysis. A different distribution of hubs between DM1 and control groups was found in the hub analysis, and differences of BC and clustering in DM1 patients compared to the controls are demonstrated at a regional level.

In the present study, we found a widespread distribution of cortical GM atrophy across all cerebral lobes. Subcortical GM loss was also detected in thalamic and basal ganglia structures. These findings are in line with previous reports describing widespread cortical and subcortical GM alterations in DM1 [7, 14–21]. No significant correlation was found between GM volumes and CTG expansion as in the previous reports [17, 20]. On the contrary, significant negative correlations were found between regional GM volumes and CTG repeat length in other studies [16, 19]. These conflicting results may be due to differences in the detailed analysis methods, the clinical variability of the study population, the somatic mosaicism of CTG repeat lengths, or repeat number increases throughout life, even in post-mitotic tissues [3, 31]. There are considerable tissue variations in repeat lengths, and repeat size in the blood might not correlate with that in the brain.

In accordance with the previous study that investigated the brain connectomics in DM1 using resting-state functional MRI and graph theoretical analysis [27], DM1 patients did not show significant differences in global network measures compared with the controls. This lack of significant reduction in global measures of connectivity is consistent with the relative preservation of general cognitive function in DM1 compared with common dementia diseases. Alzheimer disease, which is the most common type of dementia, presents with disrupted segregation and integration in brain networks and this disruption is significantly correlated with cognitive decline [32]. On the contrary, scores on overall intelligence quotient measured by the Wechsler Adult Intelligence Scale and MMSE scores are usually within the normal range in adult-onset DM1 patients [5]. Indeed, most of the DM1 patients in our study also showed scores within the normal range in MMSE.

Compensatory mechanisms explain why the efficiency of the structural network is preserved as a whole while the GM volume reduction distributes extensively. Indeed, increased BC was shown in the left fusiform gyrus, superior temporal gyrus, superior frontal gyrus, and right precuneus by comparing the DM1 network to controls. BC is regarded as a measure of integration, and a node with high betweenness is at the intersection of many short paths and likely participate in large number functional interactions in a structural network [33]. Another possible explanation for preserving the global network measurements is that a reduction of GM volume distributes to the same extent across the brain. However, while the GM volume reduction distributed extensively in DM1 in our study, there was a considerable difference in the extent of GM volume reduction for each region of the brain.

The left fusiform gyrus is one of the sites where increased BC was observed in DM1 patients and it is also the only region where a hub node with BC value over 2SD was found in hub analysis of DM1 patients. Increased BC in the left fusiform gyrus might be associated with abnormalities of face perception in DM1 patients. The fusiform gyrus includes the fusiform face area, one of the core of the face perception network. In face perception, it has been observed that the right fusiform face area is involved in holistic processing whereas the left fusiform face area is involved in feature-based (analytic) processing [34, 35]. It has been reported that DM1 patients with deficits in facial memory ability tended to employ feature-based analysis rather than holistic analysis [8].

Hemispheric lateralization in the face perception network has been reported to be due to an asymmetric interhemispheric recruitment at the level of the occipital face area [36]. Furthermore, this asymmetry recruitment was correlated with GM volume of occipital face areas in the study. In our study, regional clustering in the DM1 group was reduced in the right inferior occipital gyrus, which includes the occipital face area, and this reduction might be associated with leftward lateralization in face perception network in DM1 patients.

Increased BC in the left fusiform gyrus found in our study may also be associated with theory of mind impairment in DM1 patients. Theory of mind, which refers to the ability to infer other people’s mental status, thoughts, and feelings, is necessary to emphasize and have a good relationship with others in social situations. Previous reports have found DM1 patients showed theory of mind impairment [11, 37], and this impairment was associated with increased centrality in the left inferior temporal gyrus, including the fusiform gyrus [37].

Decreased BC was observed in the right caudate nucleus and putamen. A previous study assessing the relation between personality traits and changes to functional connectivity within the default mode network in DM1 patients has shown that decreased functional connectivity in the right caudate nucleus and putamen correlated with schizotypal-paranoid traits [38]. It was hypothesized that the relation between decreased functional connectivity in the striatum and schizotypal-paranoid traits might be explained by impairment in the cognitive flexibility.

Regarding the regional clustering in DM1 patients, decreased regions were found in the right orbitofrontal gyrus, precuneus, and left pallidum besides the right inferior occipital gyrus. The orbitofrontal gyrus has been reported to be involved in odor recognition [39, 40], and impairment in odor recognition has been described in DM1 patients [10]. However, decreased clustering may be affected by cortical thinning and its interpretation remains controversial so far. Previous reports have described reduced cortical thickness in several regions including the lateral occipital cortex and precuneus in DM1 patients [20, 41].

The limitations of our study are as follows. First, DM1 subjects represented a heterogeneous sample. Clinical variability, including age at onset and disease duration of DM1 patients, may have affected our results. Three of the four DM1 patients with decreased MMSE scores in this study had juvenile onset. Clinical presentation of childhood or juvenile onset DM1 is different from classic adult onset DM1, and CNS symptoms often predominate over muscle symptoms in the former [5]. There are a few longitudinal follow-up studies of cognitive impairment in DM1; an evolution of cognitive impairment with disease progression is suggested by studies that have shown cognitive decline in some domains over several years in DM1 [42, 43]. Second, the lack of neurocognitive and neuropsychiatric profiles’ correlation with changes in network connectivity prohibits further pathophysiological evaluations. Finally, two models of 3.0-tesla MR system were enforced in our subjects. However, to minimize the influence due to differences in MR systems, the ratio of the two MR systems in both the DM1 and the control group was equalized.

In conclusion, in contrast to the widespread reduction of GM volume on VBM, DM1 patients did not show significant differences in global network measures of graph theoretical analysis, and this result is very consistent with the preservation of general cognitive function in DM1 patients.

## Supporting information

S1 Fig Results of assessments of network resilience to random failure or targeted attack. There were no significant differences in the network resilience to either random or targeted attack between DM1 and controls.
(TIF)

S1 Table Details of the clinical characteristics of patients with myotonic dystrophy type 1. (XLSX)
