# Supplementary figures and images for: Brain gray matter structural network in myotonic dystrophy type 1
Source: PLoS One. 2017 Nov 2;12(11):e0187343. doi: 10.1371/journal.pone.0187343 (PMC5667809; doi:10.1371/journal.pone.0187343)

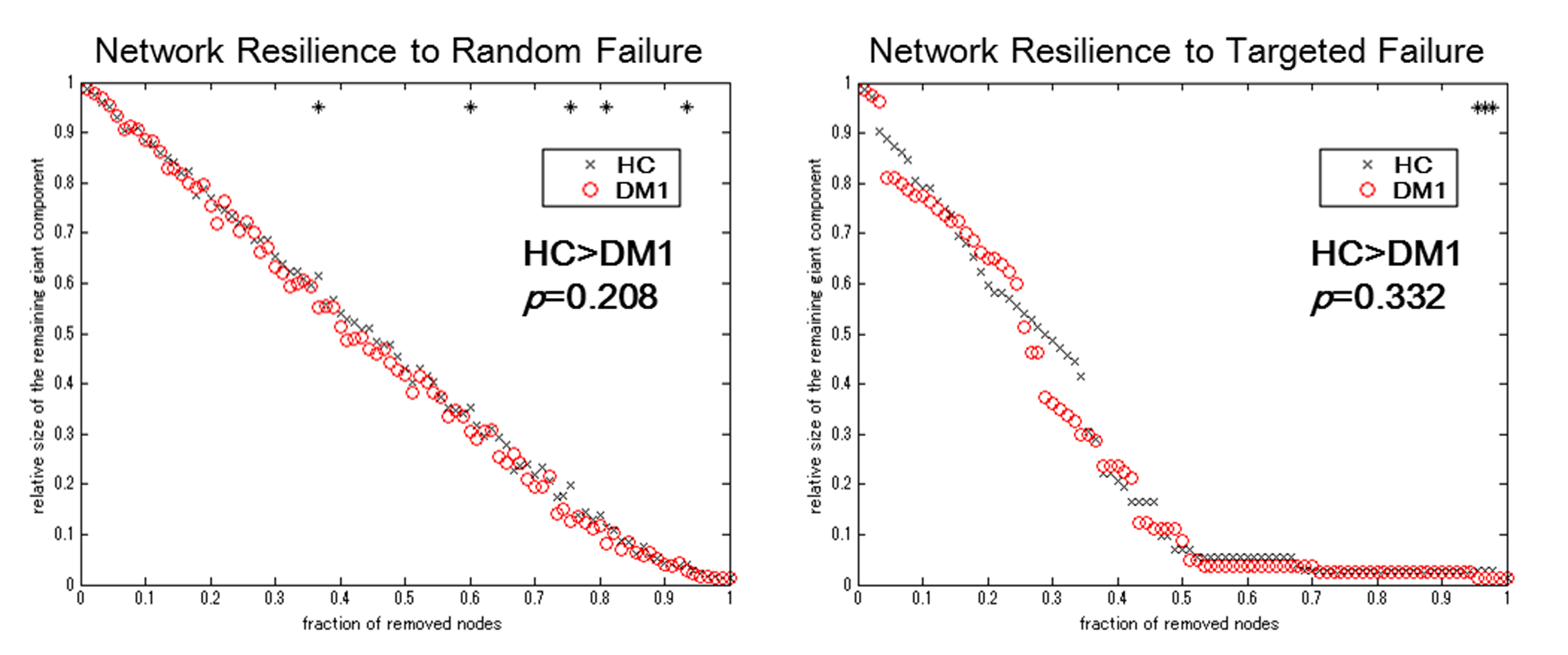

Supplement: S1 Fig — There were no significant differences in the network resilience to either random or targeted attack between DM1 and controls. (TIF) [file pone.0187343.s001.tif]
